# P-829. Words Matter: Impact of Microbiology Laboratory Result Presentation on Antibiotic Stewardship

**DOI:** 10.1093/ofid/ofaf695.1037

**Published:** 2026-01-11

**Authors:** Stephanie Harding, Nick R Harris, Christina M Brummett

**Affiliations:** Wesley Medical Center, Wichita, KS; Wesley Medical Center, Wichita, KS; Wesley Medical Center, Wichita, KS

## Abstract

**Background:**

Advancements in laboratory diagnostics are continually occurring and accuracy in interpreting results directly affects optimal patient care. In 2019, Wesley Healthcare surveyed providers on their interpretation of common microbiology results. Feedback led to several updates in how common organisms were reported. The purpose of this study was to evaluate whether these changes in reporting positively impacted clinical decisions made from the updated results.

**Figure:**
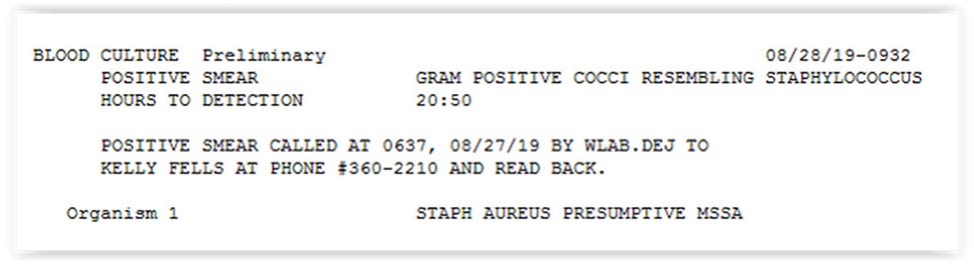
Blood cultures positive for S. aureus Pre-Intervention

**Figure:**
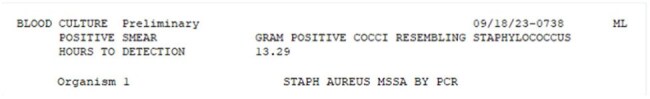
Blood cultures positive for S. aureus Post-Intervention

**Methods:**

This quasi-experimental study evaluated the impact of different verbiage on common microbiology results. A survey with six patient cases was sent to providers in 2019 and 2024. Patient cases remained the same in each survey, but utilized the current presentation of microbiology results at the time of the survey. Recipients were asked to make clinical decisions based on the microbiology results presented. The topics included de-escalation of antibiotics based on polymerase chain reaction (PCR) for positive blood cultures (Images 1 and 2), evaluation of *C. difficile* PCR and enzyme immunoassays, impact of recent immunization on *S. pneumoniae* urine antigen results, susceptibilities of Group C *Streptococcus* and *H. influenzae* (Images 3 and 4), and understanding of minimum inhibitory concentrations (MIC). Chi-squared statistics were used to compare results.

**Figure:**
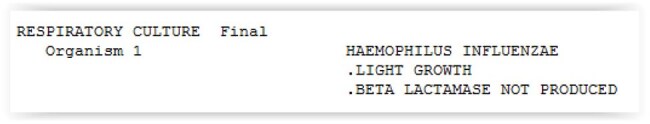
H. Influenzae Results (Pre-Intervention)

**Figure:**
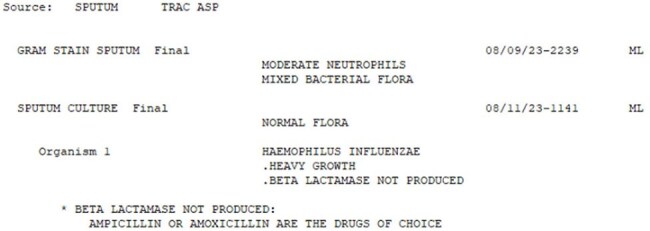
H. Influenzae Results (Post-Intervention)

**Results:**

Several trends were seen in both groups. Questions accompanied by lab results that included detailed comments had the highest percentage of correct responses. This included our *C. difficile* (Pre: 92%, Post: 98%) and *S. pneumoniae* urine antigen (Pre: 95%, Post: 94%) results. Culture results without any guidance or adjustment between surveys (Group C *Streptococcus*, Pre:72%, Post: 81%), had sustained lower rates of correct interpretation and resulted in provider reluctance to de-escalate antibiotics. Culture results which had guidance added between the two time periods had a significant increase in percentage of correct responses. This included blood cultures results by PCR (Pre: 58%, Post: 92%, p= < 0.0001) and *H. influenzae* (Pre: 86%, Post: 98%, p= < 0.02).

**Conclusion:**

Addition of detailed interpretative comments to microbiology results may lead to improved de-escalation and promote appropriate antibiotic selection.

**Disclosures:**

All Authors: No reported disclosures

